# Right ventricular outflow tract doppler flow abnormalities suggestive of pulmonary embolism – case series and review

**DOI:** 10.1186/s13089-024-00377-2

**Published:** 2024-12-18

**Authors:** Toni Ivičić, Jasmin Hamzić, Bojana Radulović, Ivan Gornik

**Affiliations:** Emergency Department, University Hospital Centre, Zagreb, Croatia

**Keywords:** Early systolic notching, Right ventricular outflow tract, Pulmonary embolism, Focused echocardiography, Emergency medicine, Pulsed wave doppler

## Abstract

**Background:**

Pulmonary embolism (PE) is one of the most challenging diagnoses in emergency medicine, mainly because symptoms range from asymptomatic disease to sudden death. The role of echocardiography in the workup of suspected PE has been supportive and used primarily to assess the right ventricular (RV) size and function, which is important for risk stratification. Several echocardiographic parameters described in the literature lack the desired accuracy. Recently, a potential value of less well-recognized RV outflow tract (RVOT) Doppler variables has been reported. The early systolic notching (ESN) pattern was observed in 92% of patients with high and intermediate risk PE, making it a promising sign in selected PE patients.

**Case presentation:**

In this case series, we demonstrate a typical ESN pattern on RVOT Doppler evaluation in three patients with intermediate-risk PE presenting to our emergency department (ED). None of the patients had been previously diagnosed with pulmonary hypertension or other chronic pulmonary and cardiac disease. The pre-test probability was low. Massive proximal emboli were found on CT angiograms, involving pulmonary truncus or main pulmonary arteries. Previously, the ESN pattern was identified on a focused echocardiogram, which was the only echocardiographic indicator of increased pulmonary vascular resistance.

**Conclusions:**

RVOT Doppler flow pattern of ESN has potential clinical utility for the detection of PE in ED patients. ESN could identify patients at higher risk, which are otherwise stratified as low risk according to the latest guidelines. Moreover, this case series illustrates that even in the absence of other echocardiographic findings of RV strain, the presence of ESN should alert to the possibility of acute PE. Further prospective studies are needed to assess its diagnostic value in a selected subgroup of patients, similar to the cases presented, that would have no other obvious reason for the altered RVOT Doppler curve.

## Background

Venous thromboembolism (VTE), presenting as deep vein thrombosis (DVT) or pulmonary embolism (PE), is the third most frequent acute cardiovascular syndrome, behind myocardial infarction and stroke [1]. Over time, a rising annual PE incidence in European countries has been reported in numerous studies, which is mostly explained by the widespread adoption of CT pulmonary angiography (CTPA) for diagnosing PE in 1998 [2–4]. Although it led to an 80% increase in PE incidence in the next 8 years, an expected change in PE mortality was not observed. There is a tendency towards overdiagnosis of subsegmental or non-existent PE in the modern era, which led to a false drop in case fatality rates [5, 6]. However, the increased use of more effective therapies and interventions, summarized in leading European and American guidelines, has most likely exerted a positive effect on PE outcomes [7, 8].

Contrast CTPA, and to a lesser extent ventilation-perfusion scan, remains the diagnostic modality of choice for the diagnosis of PE. Its high spatial and temporal resolution allows adequate visualization of the pulmonary arteries down to the subsegmental level [9, 10]. Significant radiation exposure, especially to young female breast tissue, and the tendency to overuse because of easy accessibility, remain its main weaknesses [11].

On the other hand, the role of echocardiography in the workup of suspected PE has been supportive and used primarily to assess right ventricular (RV) size and function, which is important for risk stratification of PE to high, intermediate-high, intermediate-low and low risk, according to ESC guidelines [7]. Several echocardiographic parameters described in the literature, including McConnell`s sign, the 60/60 sign and decreased tricuspid annular plane systolic excursion (TAPSE), lack the desired sensitivity and negative predictive value to be used in diagnostic algorithms for suspected PE [12, 13].

Recently, a potential value of less well-recognized RV outflow tract (RVOT) Doppler variables has been reported in patients with high and intermediate risk PE (massive and submassive PE, respectively). Early systolic notching pattern (ESN) was observed in 92% of patients with MPE or SMPE, making it a promising non-invasive sign in selected PE patients, with good specificity and positive predictive value [14].

In this case series, we demonstrate a typical ESN pattern on RVOT Doppler evaluation in three patients with intermediate-risk PE presenting to our emergency department (ED) during the 1 month. None of the patients had been previously diagnosed with pulmonary hypertension or other chronic pulmonary and cardiac disease. Massive proximal emboli were found on CT angiograms, involving pulmonary truncus or main pulmonary arteries. Previously, the ESN pattern was identified on a focused echocardiogram. Written consent was obtained for all patients.

## Case presentations

### Case 1

**Fig. 1:**
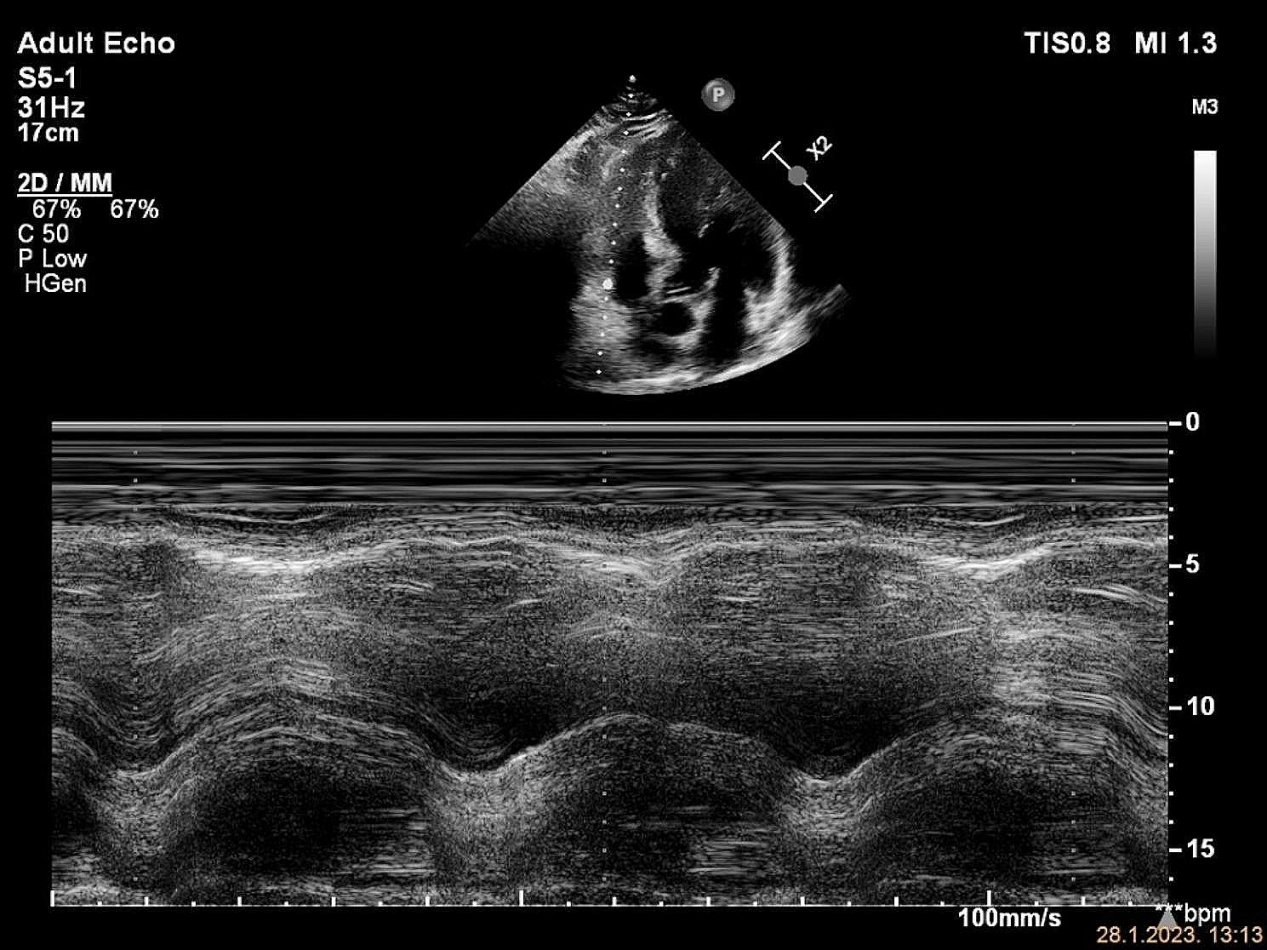
Normal RV: LV ratio, TAPSE 23 mm

**Fig. 2:**
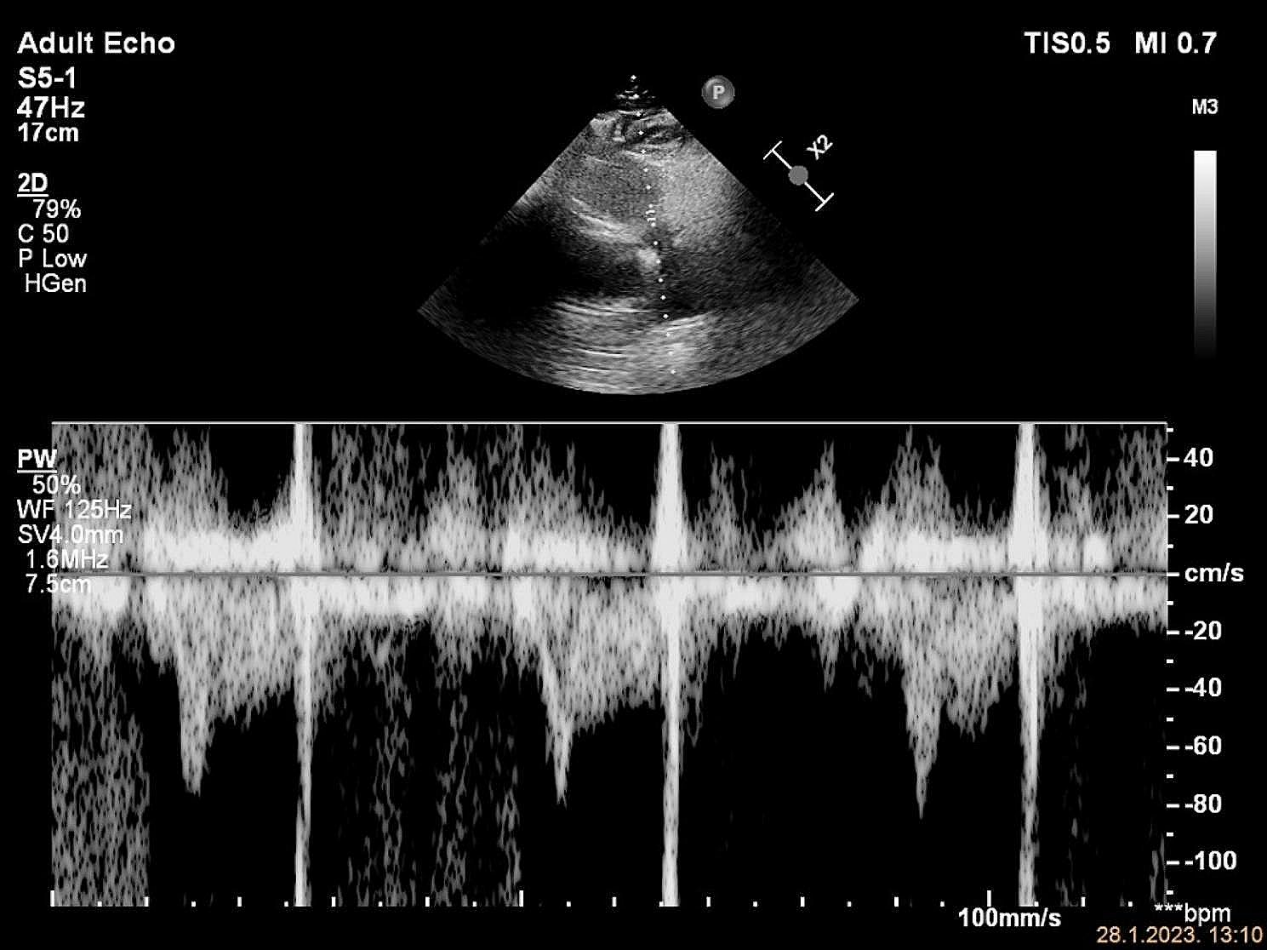
RVOT VTI early systolic notch (ESN) pattern, AT 45 ms

A 59-year-old male patient presented to the ED with left calf swelling and mild exertional dyspnea. He complained of a dry cough with pleuritic and stabbing chest pain. Unilateral leg swelling was worsening during the previous 14 days, without signs of skin infection. The patient denied any past medical history, except for arterial hypertension. Family history was positive for venous thromboembolism. On physical examination, the patient`s vital signs were normal, including blood pressure of 140/80 mmHg and heart rate of 80/min, without signs of arrhythmia. Pulse oximetry (SpO2) showed 95% hemoglobin saturation on room air. Lung auscultation revealed normal breath sounds. Lower extremities were asymmetric with mild pitting edema of the left calf. A compression ultrasound of the left leg was immediately performed, which showed DVT of the common and superficial femoral vein. Because of the dyspnea and chest pain, a focused echocardiogram was performed for the signs of RV dysfunction. It showed no RV dilation, normal RV: LV ratio of 1:2 and normal free wall and apical RV contractility. TAPSE measured 23 mm (Fig. 1). Inferior vena cava (IVC) diameter was 15 mm with 40% respiratory variability. Tricuspid regurgitant (TR) jet was not identified, so TR Vmax and pressure gradient (PG) could not be measured. RVOT VTI had decreased acceleration time (AT) of 45 msec, with an ESN pattern (Fig. 2).

**Fig. 3:**
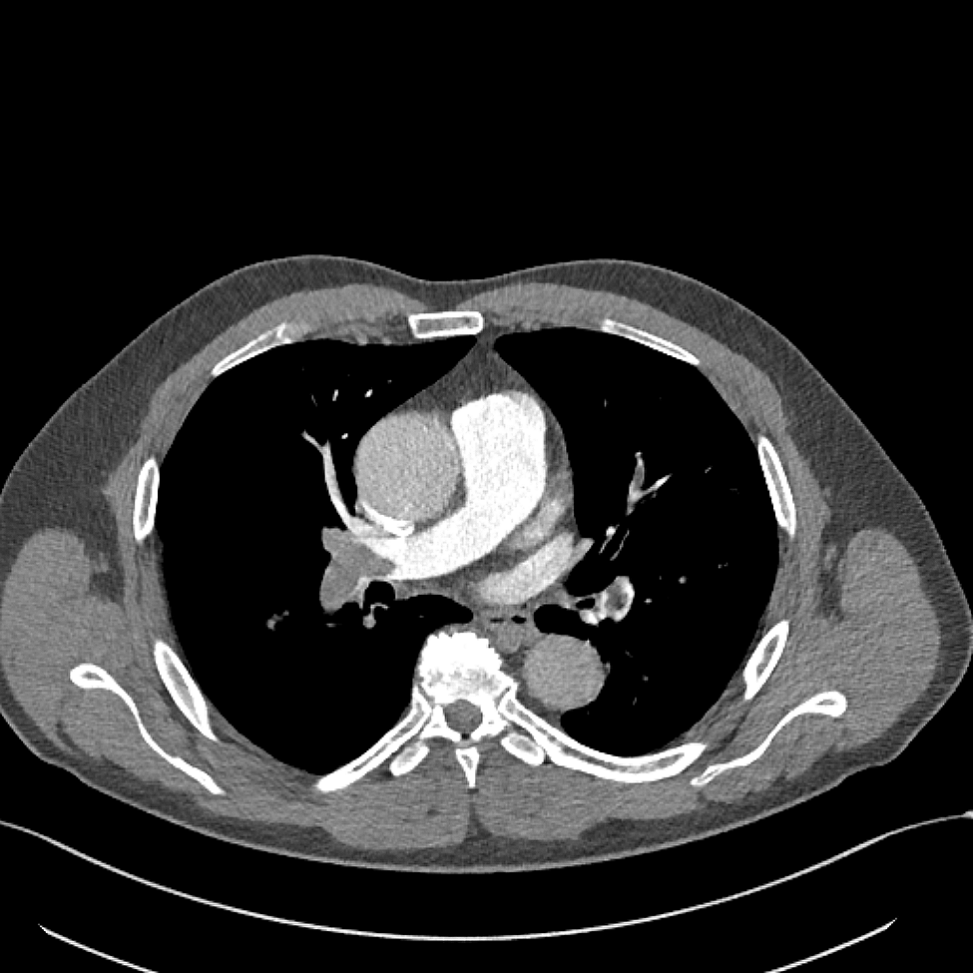
Massive embolus in the right main pulmonary artery and its branches

**Fig. 4:**
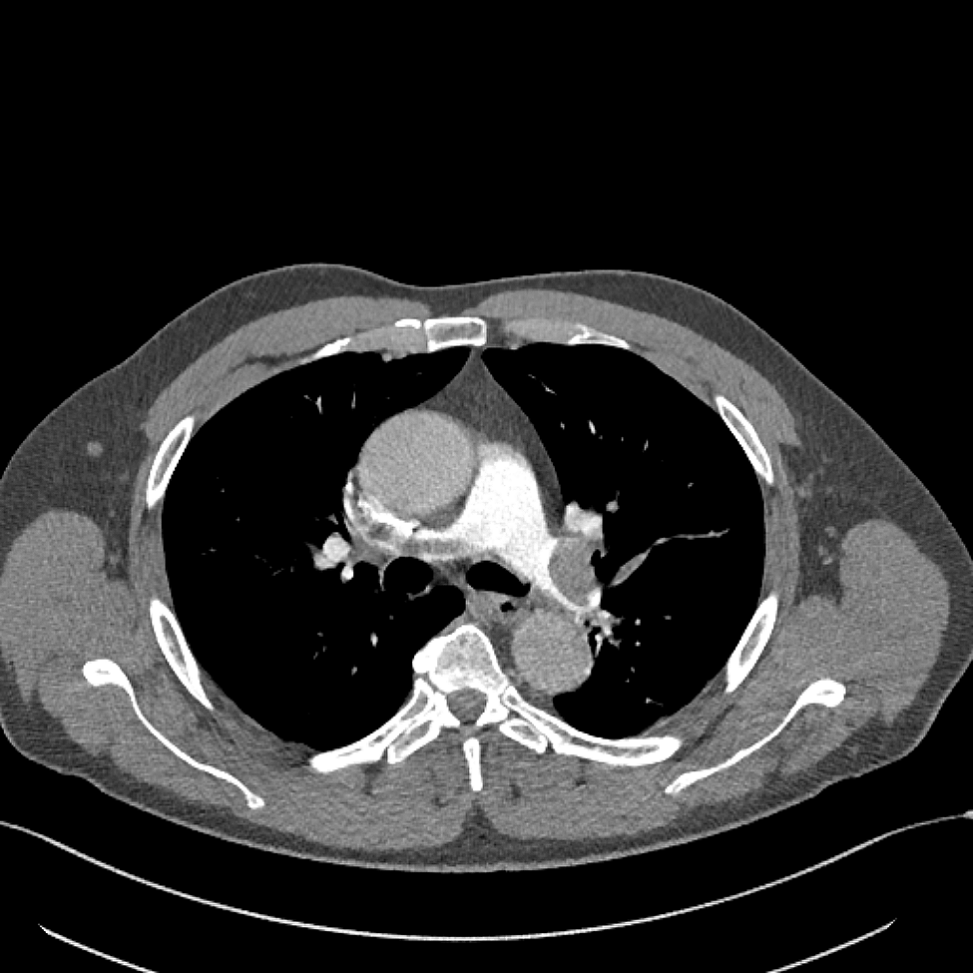
Massive embolus in the left main pulmonary artery and its branches

Treatment with low molecular weight heparin in therapeutic doses was started immediately. Electrocardiogram (ECG) showed no signs of RV strain. Laboratory findings were normal, including cardiac biomarkers – hsTnI (< 5.0 ng/L) and NTproBNP (24 ng/L). CTPA was performed and showed extensive thromboembolism in both main pulmonary arteries and its lobar and segmental branches (Figs. 3 and 4).

Pulmonary embolism severity index (PESI) was low risk (69 pts), but because of the signs of RV dysfunction (ESN, AT 45 ms), the patient was risk stratified as intermediate-low risk, according to ESC guidelines.

The patient was admitted to the cardiology ward and eventually discharged from the hospital in good health without adverse events.

### Case 2

A 58-year-old female presented to our ED with shortness of breath, mostly in exertion, malaise and exercise intolerance for the past 2 months. She tolerated lying flat and denied leg swelling. There were no complaints of respiratory tract infection symptoms. Her past medical history revealed arterial hypertension treated with oral antihypertensives. Family history was not significant. On physical examination, blood pressure was normal, 150/100 mmHg, with a normal heart rate of 83/min. SpO2 was 94% without dyspnea at rest. The patient had normal breath and heart sounds on auscultation. There were no signs of congestion and the lower extremities were symmetrical. ECG showed T wave inversion in leads V1-V4. Chest X-ray had no pneumonic infiltrates or signs of pulmonary congestion. Among laboratory results worth noting, d-dimers were elevated, 4.46 mg/L FEU, hsTnI was normal and NTproBNP came back slightly elevated, 605 ng/L.

**Fig. 5:**
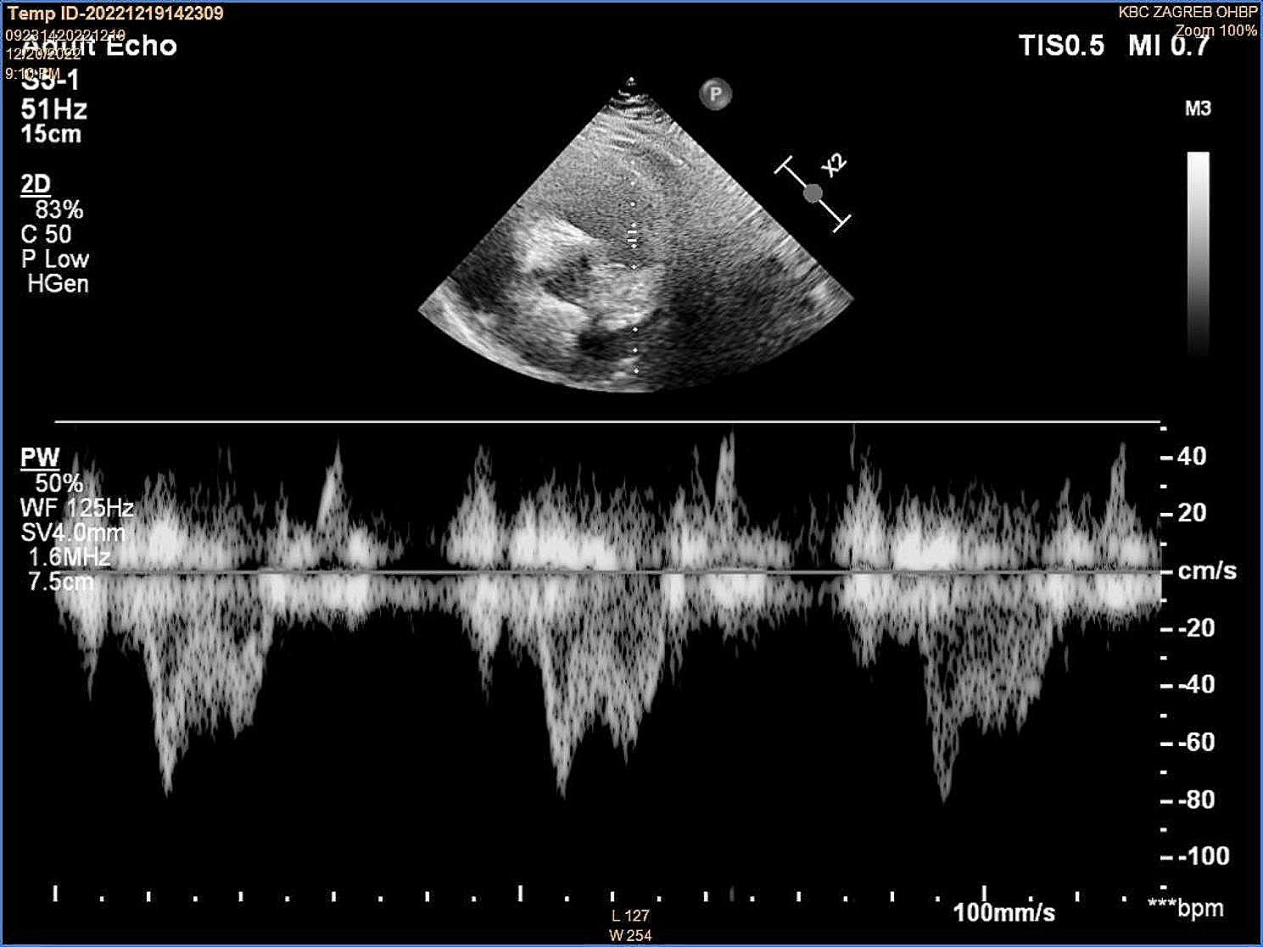
RVOT VTI early systolic notch (ESN) pattern, AT 55 ms

A focused echocardiogram was performed which showed a normal-sized RV, and a normal RV: LV ratio of 0.7. RV contractility was not altered, with TAPSE measuring 24 mm. TR jet was insufficient for TR PG estimation. RVOT Doppler evaluation revealed an ESN pattern of velocity time integral and decreased AT 55 msec (Fig. 5).

**Fig. 6:**
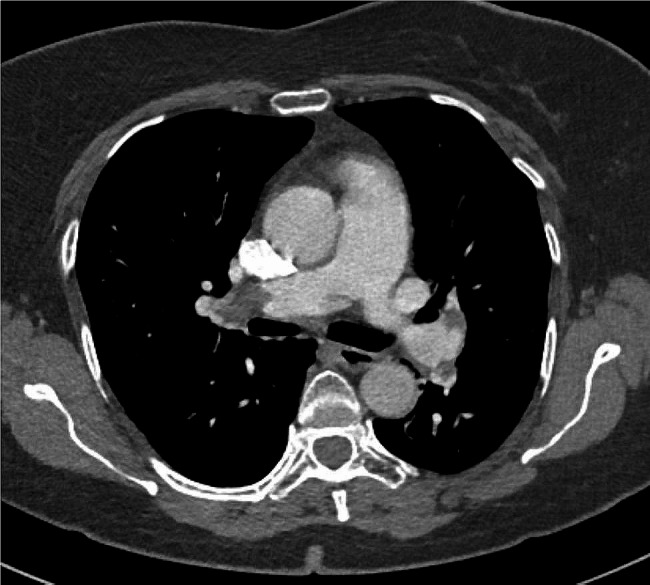
Massive filling defects in right main pulmonary artery

CTPA, done afterward, showed massive filling defects in the right main pulmonary artery and lobar artery for the left inferior lobe (Fig. 6).

PESI score predicted very low risk (58 pts), up to 1.6% for 30-day mortality, but because of elevated cardiac biomarkers and echocardiographic signs of increased RV afterload, the patient was risk stratified as intermediate-high risk according to ESC guidelines and admitted to the cardiology ward.

### Case 3

A 45-year-old male was admitted to the emergency department with right calf pain in the past three days. He didn’t notice swelling of the right leg, but he complained of chest pain during the night and mild shortness of breath in exertion. Recently he had a long transatlantic air flight. Past medical history was insignificant except for regular alcohol drinking during the past 18 months. Family history was negative for thromboembolic disease. Vital signs were normal, with blood pressure 140/90 mmHg, regular 75 heartbeats per minute and SpO2 99% on pulse oximetry. Physical examination revealed a right calf circumference difference of 4 cm compared to the left calf. Lung and cardiac physical were insignificant. A compression ultrasound of the left leg was performed afterward, which showed DVT of the superficial femoral vein below the great saphenous vein inlet, spreading distally to the popliteal vein.

**Fig. 7:**
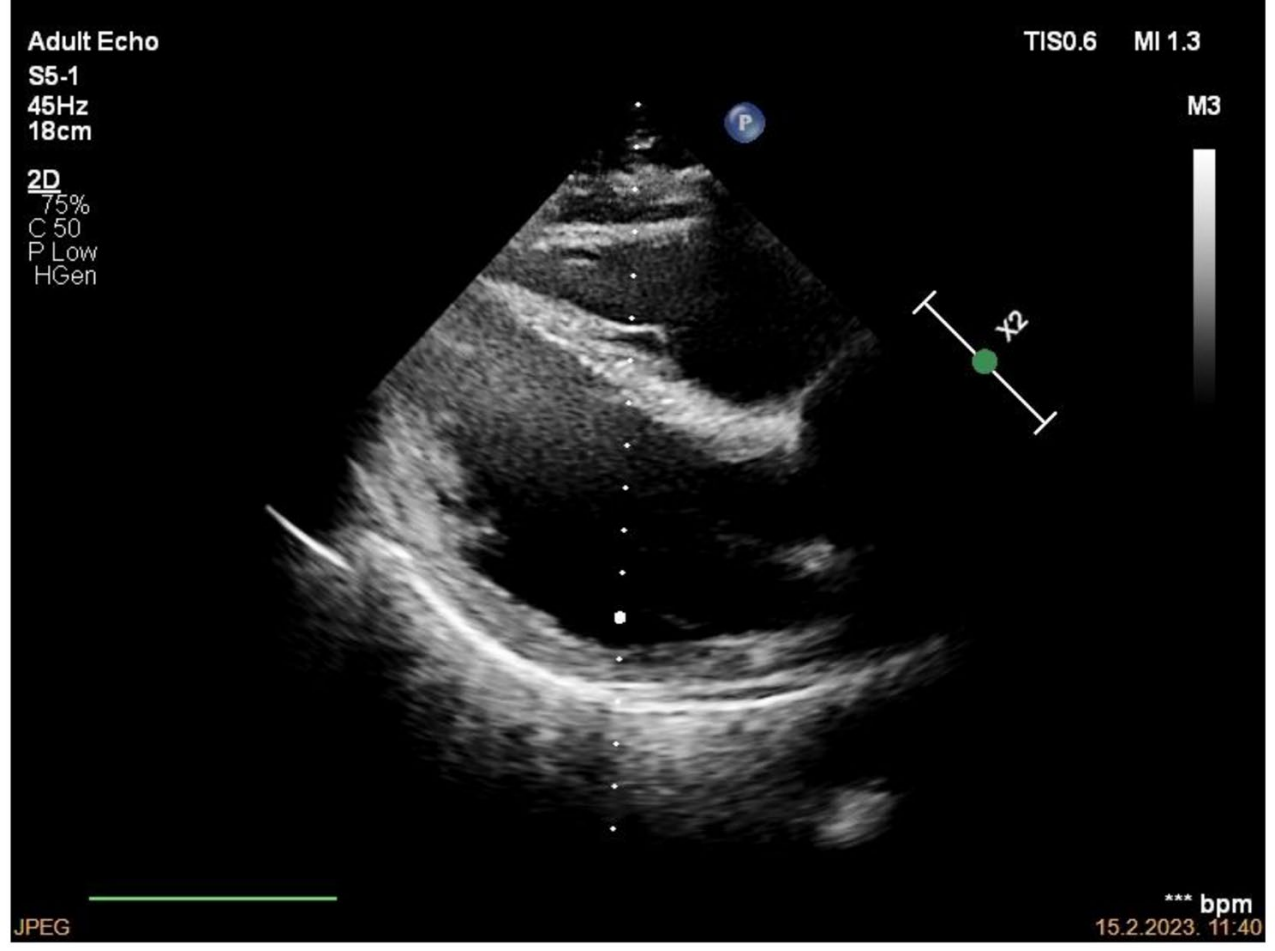
Normal RV size and RV: LV ratio

**Figs. 8:**
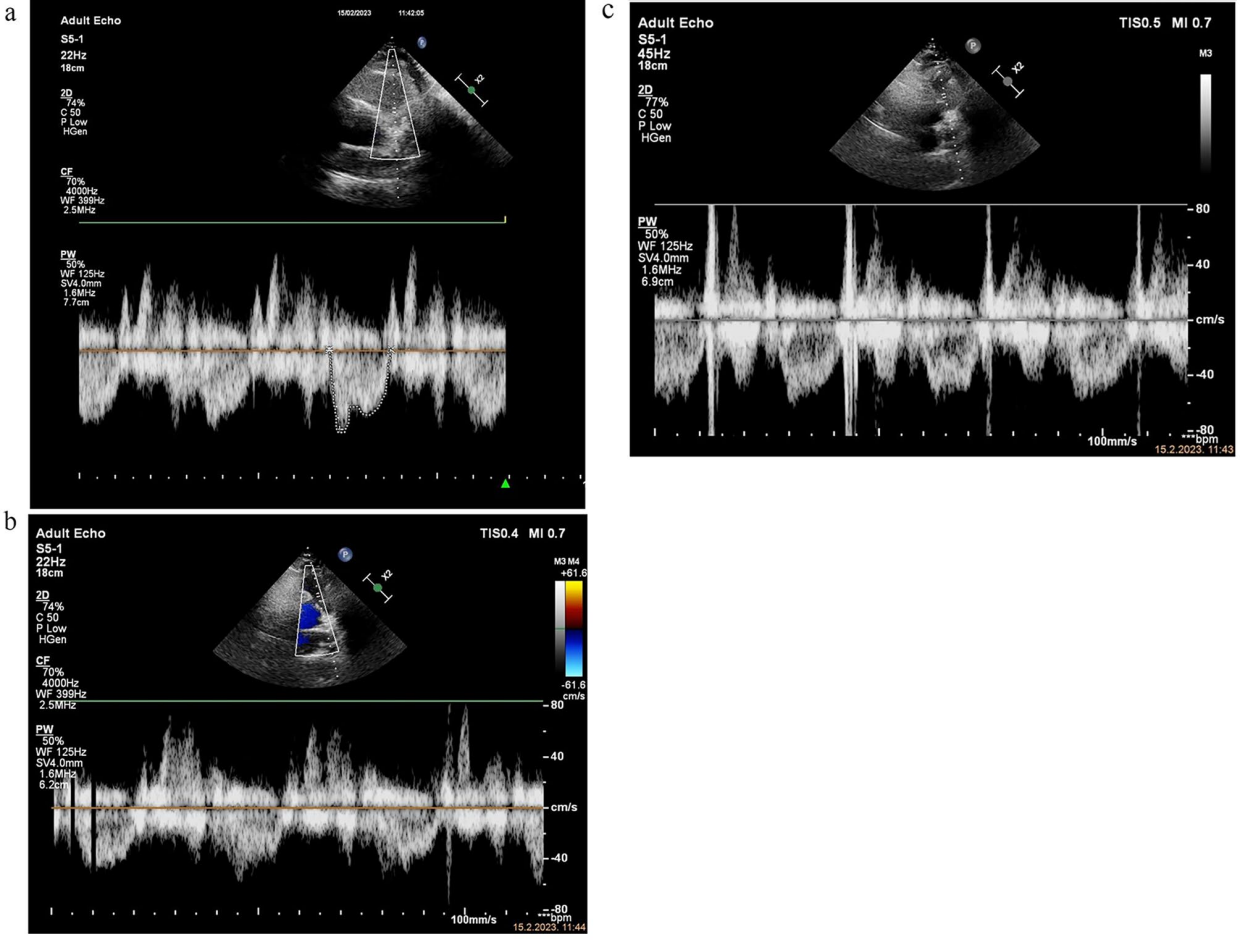
8a, b, c, ESN pattern of RVOT VTI and reduced AT

A focused right heart echocardiogram revealed a normal-sized RV and normal RV: LV ratio (Fig. 7). RV free wall and apical contractility were normal, TAPSE measuring 23 mm. Tricuspid regurgitant jet was insufficient for sPAP estimation. IVC diameter, measured in the longitudinal plane, was 11 mm with adequate variability with respiration. RVOT pulsed wave Doppler trace showed ESN and reduced acceleration time of 60 ms. Estimated mPAP was 53 mmHg (Fig. 8).

**Fig. 9:**
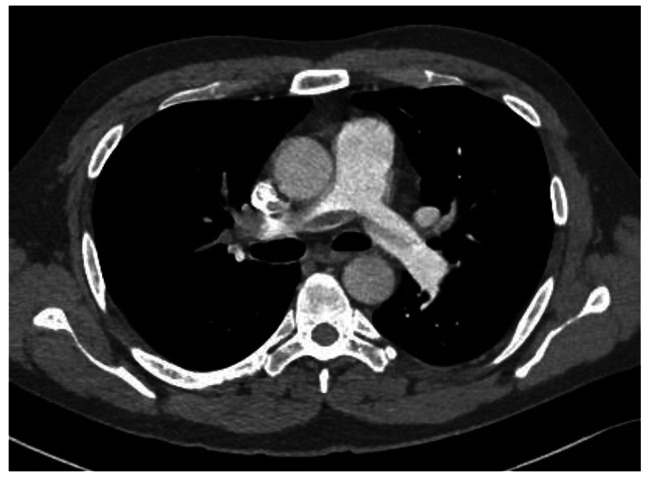
Saddle pulmonary embolus at pulmonary truncus bifurcation and main pulmonary arteries

CTPA was performed due to high suspicion of pulmonary embolism, which showed saddle pulmonary embolus at pulmonary truncus bifurcation, involving both main pulmonary arteries (Fig. 9). Laboratory results were insignificant, including normal hs-TnI and NTproBNP (hsTnI < 5.0 ng/L and 321 ng/L, respectively).

PESI score of 55 pts predicted very low risk. There were no laboratory signs of RV strain, but because of echocardiographic findings (ESN, AT 60 ms), he was risk stratified as intermediate-low risk, according to ESC guidelines.

## Discussion

Echocardiography has traditionally had a supportive role in the evaluation of patients with suspected PE. It is primarily used to stratify the early risk of patients with PE, i.e., for evaluation of RV size and function [15]. Numerous echocardiographic parameters, including McConnel`s sign, the 60/60 sign (AT < 60 ms, TR PG > 30 mmHg, but < 60 mmHg), septal wall flattening, RV dilation, etc., lack the desired sensitivity and specificity compared to CTPA for the diagnosis of PE in a broader population. Because of the reported negative predictive value of 40–50%, a negative result cannot exclude PE in unselected patients [13]. Pulmonary ejection acceleration time less than 60 ms combined with a peak systolic tricuspid valve gradient < 60 mmHg (‘60/60’ sign) and with McConnell sign, appears to have the best specificity of 94%. Unfortunately, these findings are present in only 12 to 20% of PE patients and cannot exclude PE [16]. Regarding risk assessment, an RV/LV diameter ratio > 1.0 and a TAPSE < 16 mm have most frequently been associated with an unfavorable prognosis [17].

On the other hand, signs of RV overload or dysfunction may also be found in the absence of acute PE and may be due to concomitant cardiac or respiratory disease. According to Bova et al., adding echocardiography to the diagnostic strategy for PE would avoid about 12 to 28% of lung-scan angiography procedures, but would cause inappropriate treatment of 4 to 14% of all treated patients [18].

In the study conducted by Afonso et al., early systolic notching was reported with high specificity for the diagnosis of massive or submassive PE (high risk or intermediate risk, respectively) in a limited population. This study excluded patients with known pulmonary hypertension. ESN was observed in 92% of patients with MPE or SMPE. On the other hand, it was found in only 2% of patients with subsegmental PE and no patients without PE [14]. More recently, another multicentric study aimed to determine the diagnostic accuracy of ESN for PE in emergency department patients. In all ED patients, the sensitivity of ESN for PE was 34%, and the specificity was 97% [19].

The pathophysiologic rationale behind ESN is compatible with the concept of a pressure wave reflected from the pulmonary arteries [20]. Under normal circumstances, a reflected pressure wave returns after the RV has completed its ejection, meaning an optimal ventricular–vascular coupling [21]. Pulmonary hypertension, by increasing both the velocity and amplitude, causes an earlier return of reflected wave, manifesting as mid-systolic deceleration on the flow-velocity curve. Time-to-notch might be affected by the site of vascular changes in patients with precapillary pulmonary hypertension. In the presence of proximal thromboemboli, pressure waves return very early, resulting in an early notch. In the study by Torbicki et al., despite a relatively mild increase in systolic pulmonary artery pressure, indicated by TR PG, the most disturbed flow-velocity curves were found in patients with acute PE. In other words, notching is strongly correlated with pulmonary vascular resistance (RV afterload), but may be dissociated from pulmonary artery pressure in patients with acute PE. The presence of proximal thromboemboli, and not the severity of pulmonary hypertension, seemed to be the major determinant of RVOT flow-velocity curve ESN [22].

This was the case with all of the three patients presented in this case series. Large proximal emboli, involving pulmonary truncus or main pulmonary arteries, led to early pressure wave reflection, which altered RV-pulmonary artery coupling. None of the patients had prior pulmonary arterial hypertension or cardiac disease that would explain increased RV afterload and all other echocardiographic markers of RV dysfunction were negative, except ESN on RVOT Doppler. Interestingly, even though large emboli were found on CTPA, all the patients were hemodynamically stable, i.e. without signs of obstructive shock and not requiring thrombolytic therapy. Moreover, because of low PESI and sPESI scores, according to ESC guidelines, all the patients would be classified as low risk. However, signs of RV dysfunction on echocardiography or elevated cardiac biomarker levels may be present, despite a low-risk PESI. Guidelines suggest these patients should be classified into the intermediate-risk category, because such discrepancies and their implications for the management of PE are not yet fully understood [7]. That is how the patients in this case series were risk classified by treating physicians, even though among the signs of RV pressure overload, listed in the guidelines, ESN is not specifically noted. In addition, criteria for other signs, including 60/60 sign, were not met by any patient.

Given the high specificity of ESN for the diagnosis of PE, reported in the two studies mentioned above, there is a potential to bypass CTPA in diagnostic algorithm in carefully selected patients. Further research is warranted in a more defined subgroup of patients, similar to the cases presented, that would have no other obvious reason for altered RVOT flow-velocity curve, for example in patients younger than 60 years old, without prior pulmonary hypertension, cardiac disease or malignant disease. The specificity and positive predictive value of ESN in these circumstances could be comparable to that of CTPA. Starting therapeutic anticoagulation, while avoiding radiation exposure, could have the most beneficial effects in this very subgroup of younger patients. Significant radiation exposure during CTPA, especially to young female breast tissue, remains one of its main adverse effects.

Until additional validated data becomes available, echocardiography should not be used as the primary screening test or a gatekeeper for CTPA in the diagnosis of acute PE.

## Conclusion

RVOT Doppler flow pattern of ESN has potential clinical utility for the detection of PE in ED patients. ESN could identify patients at higher risk, which are otherwise stratified as low risk according to the latest guidelines. The presence of ESN could be the only echocardiographic finding of RV dysfunction, which should alert to the possibility of acute PE. Further prospective studies are needed to assess its diagnostic value in selected subgroup of patients.

## Data Availability

The data regarding this case series is available in the Hospital`s Information System (BIS).

## Abbreviations

AT: Acceleration time
CTPA: Computed tomography pulmonary angiography
DVT: Deep vein thrombosis
ECG: Electrocardiogram
ED: Emergency department
ESC: European Society of Cardiology
ESN: Early systolic notch
IVC: Inferior vena cava
mPAP: Mean pulmonary artery pressure
MPE: Massive pulmonary embolism
PE: Pulmonary embolism
PESI: Pulmonary embolism severity index
RV: Right ventricle
RVOT: Right ventricular outflow tract
SMPE: Submassive pulmonary embolism
sPAP: Systolic pulmonary artery pressure
TAPSE: Tricuspid annular plane systolic excursion
TR PG: Tricuspid regurgitation pressure gradient
VTE: Venous thromboembolism
